# Pheromone Receptor Knock-Out Affects Pheromone Detection and Brain Structure in a Moth

**DOI:** 10.3390/biom12030341

**Published:** 2022-02-22

**Authors:** Fotini Koutroumpa, Christelle Monsempès, Sylvia Anton, Marie-Christine François, Nicolas Montagné, Emmanuelle Jacquin-Joly

**Affiliations:** 1 Institute of Ecology and Environmental Sciences of Paris, INRAE, Sorbonne Université, CNRS, IRD, UPEC, Université de Paris, 78000 Versailles, France; foteini.koutroumpa@inrae.fr (F.K.); christelle.monsempes@inrae.fr (C.M.); marie-christine.francois@inrae.fr (M.-C.F.); nicolas.montagne@sorbonne-universite.fr (N.M.); 2 INRAE, Université de Tours, ISP, 37380 Nouzilly, France; 3 Institute for Genetics, Environment and Plant Protection, INRAE, Institut Agro, Université Rennes 1, 49045 Angers, France; sylvia.anton@inrae.fr

**Keywords:** pheromone receptor, CRISPR/Cas9, macroglomerular complex, *Spodoptera littoralis*

## Abstract

Sex pheromone receptors are crucial in insects for mate finding and contribute to species premating isolation. Many pheromone receptors have been functionally characterized, especially in moths, but loss of function studies are rare. Notably, the potential role of pheromone receptors in the development of the macroglomeruli in the antennal lobe (the brain structures processing pheromone signals) is not known. Here, we used CRISPR-Cas9 to knock-out the receptor for the major component of the sex pheromone of the noctuid moth *Spodoptera littoralis*, and investigated the resulting effects on electrophysiological responses of peripheral pheromone-sensitive neurons and on the structure of the macroglomeruli. We show that the inactivation of the receptor specifically affected the responses of the corresponding antennal neurons did not impact the number of macroglomeruli in the antennal lobe but reduced the size of the macroglomerulus processing input from neurons tuned to the main pheromone component. We suggest that this mutant neuroanatomical phenotype results from a lack of neuronal activity due to the absence of the pheromone receptor and potentially reduced neural connectivity between peripheral and antennal lobe neurons. This is the first evidence of the role of a moth pheromone receptor in macroglomerulus development and extends our knowledge of the different functions odorant receptors can have in insect neurodevelopment.

## 1. Introduction

Sex pheromones represent crucial signals in intraspecific communication between individuals of the opposite sex. They play essential roles in mate finding and thus efficient reproduction and contribute to species premating isolation and eventually speciation [1,2]. In most moth species, females release a species-specific sex pheromone blend that attracts males at distance [3]. The different components of the pheromone blend are detected at the level of the main peripheral olfactory organs, the antennae, by dedicated pheromone-sensitive olfactory sensory neurons (OSNs) [4]. OSNs are housed in different types of cuticular structures called sensilla, and in moths, the long trichoid sensilla are dedicated to pheromone detection [5]. To detect pheromone molecules, the pheromone-sensitive OSNs express pheromone receptors (PRs) in their dendritic membrane [6]. These PRs constitute specialized subfamilies of odorant receptors (ORs). As with other ORs, PRs are seven-transmembrane proteins and are proposed to function as ion channels by forming a complex with a highly conserved and broadly expressed odorant receptor co-receptor (Orco) [7,8,9,10]. Usually, pheromone sensitive OSNs express only one type of PR that determines the OSN selectivity and specificity [5]. All OSNs expressing the same OR type project their axons in the same compartment in the antennal lobe (AL) called glomerulus, where they synapse with second-order neurons. In male moths, the glomeruli dedicated to sex pheromone signal integration have a larger volume than ordinary glomeruli and gather in a specific AL area called the macroglomerular complex (MGC) [11]. These macroglomeruli are easily identifiable between individuals of the same species and sex due to their shape, size and location close to the entrance of the antennal nerve [12]. The size of glomeruli within the antennal lobe is, however, subjected to plasticity as a function of for example age, rearing conditions and experience [13,14].

Many PRs have been identified and functionally characterized in moths, mainly via heterologous expression in different systems such as cell cultures, *Xenopus* oocytes, and *Drosophila* OSNs [6]. However, these systems remain heterologous and may not confidently reflect the PR function in vivo. The recent development of genome editing tools and their application to insects now allows linking gene loss with specific phenotypes [15,16]. For instance, CRISPR-Cas9 has been largely applied to knock-out Orco in different insect species, revealing its primordial function in olfactory transduction, including sex pheromone sensing in moths [17,18,19], but also in the development and maintenance of AL structure, depending on the species. In ants and bees, Orco appears to play a role in OSN development and glomerulus formation [20,21,22], whereas in *Drosophila*, loss of function of Orco does not affect the glomeruli formation in the AL [7,23].

Yet, there are only very few examples of successful PR knock-out in moths, such as *Bombyx mori*, *Helicoverpa armigera*, and *Spodoptera littoralis* [24,25,26]. The resulting effects on peripheral sensing and mating behavior have been investigated, revealing that deletion of only one PR could lead to pheromone anosmia and mating disruption, confirming that sex pheromone detection follows a dedicated labeled line pathway. However, the effect of PR knock-out on the moth MGC structure has not yet been investigated, and the function of PRs, if any, in MGC development is not known.

In this study, we took advantage of a PR knock-out line we have generated in the noctuid moth *S. littoralis* (pheromone receptor SlitOR5) [26] to investigate the peripheral effects at the OSN level and the central effects at the macroglomerulus level. We have previously shown that SlitOR5 knock-out completely abolished the global antennal response to the main component of the female sex pheromone blend, (*Z,E*)-9,11-tetradecadienyl acetate (*Z*9,*E*11-14:OAc), and affected all steps of the male courtship behavior [26]. When heterologously expressed in *Drosophila* OSNs and *Xenopus* oocytes, SlitOR5 was specifically tuned to *Z*9,*E*11-14:OAc. However, the effect of SlitOR5 knock-out was not investigated at the individual OSN level nor at the AL level. Here, we revealed that SlitOR5 loss of function impacted the activity of only one out of three characterized pheromone-sensitive OSN classes, allowing us to clearly link one OSN type activity to PR expression. We also showed that SlitOR5 knock-out led to a reduced size of the glomerulus processing information on the main pheromone component in the MGC.

## 2. Materials and Methods

### 2.1. Animal Rearing, Generation of SlitOR5 Mutants

*S. littoralis* were reared in the laboratory on a semi-artificial diet [27] at 22 °C, 60% relative humidity, and under a 16 h light:8 h dark cycle. Males and females were sexed as pupae and further reared separately since it has been shown that smelling the sex pheromone can impact chemosensory gene expression and macroglomerulus size in this species [14]. The generation of the CRISPR-Cas9 SlitOR5 knock-out (KO) homozygote individuals has been described previously [26]. Briefly, freshly laid eggs were injected with a mix of a guide RNA (AGCATAAATACTGGACCCAG TGG) designed against the first exon of the *SlitOr5* gene and the Cas9 protein. Individuals were genotyped at each generation using PCR on genomic DNA extracted from larvae pseudopods (Wizard Genomic DNA Purification Kit, Promega, Madison, WI) using gene-specific primers (*Or5*_forward: 5′-CCAAAAGGACTTGGACTTTGAA-3′; *Or5*_reverse: 5′-CCCGAATCTTTTCAGGATTAGAA-3′) encompassing the target sequence. Mutagenic events were detected by sequencing the amplification products (Biofidal, Vaulx-en-Velin, France), and G0 individuals carrying a single mutagenic event were crossed with wild-type individuals. Homozygous G2 KO individuals were obtained by crossing G1 heterozygous males and females.

### 2.2. Single-Sensillum Recordings

Single-sensillum extracellular recordings (SSRs) on three functionally characterized types of OSNs from *S. littoralis* male long trichoid (LT) sensilla (namely LT1a, LT2a, and LT2b) [28,29,30] were performed as previously described [30]. Briefly, 1-to-3 day-old males were restrained in a Styrofoam block, and the antenna was visualized under an MZ16 stereomicroscope (Leica, Wetzlar, Germany). Tungsten electrodes were inserted at the base of the sensilla using a PatchStar micromanipulator (Scientifica, Uckfield, UK). The OSNs were stimulated with an air pulse of 200 ms (0.2 L·min^−1^), odorized using a stimulus cartridge containing a filter paper loaded with 1 μg of the following pheromone components: the *S. littoralis* major pheromone component (*Z,E*)-9,11-tetradecadienyl acetate (*Z*9,*E*11-14:OAc), the minor component (*Z,E*)-9,12-tetradecadienyl acetate (*Z*9,*E*12-14:OAc) and the behavioral antagonist *Z*-9-tetradecenol (*Z*9-14:OH) (diluted at 1 μg/μL in hexane). Hexane was used as a control, and each assay was repeated 12 times on KO and 13 times on wild-type males. Pheromone compounds were either synthesized in the lab or purchased from Pherobank (Wijk bij Duurstede, The Netherlands). Hexane was purchased from Carlo Erba Reagents (Val de Reuil, France). The electrical signal was amplified, high-pass (1 Hz), and low-pass (3 kHz) filtered using a CyberAmp 320 (Molecular Devices, Sunnyvale, CA, USA) and sampled at 10 kHz via a Digidata 1440A acquisition board (Molecular Devices). Recordings and analyses were performed with pCLAMP™ 10 (Molecular Devices).

### 2.3. Brain Dissection and Immunostaining

The structure of brain neuropil, and specifically the antennal lobe MGC of *S. littoralis* males, was revealed by using immunostaining with an antibody against the *Drosophila* vesicle-associated protein synapsin 1 (SYNORF1, Developmental Studies Hybridoma Bank, University of Iowa, Iowa City, IA, USA). This staining method has been used previously to reveal AL glomeruli in the same moth species [14,31]. Briefly, brains from 12 wild type and 12 KO newly emerged virgin and naïve males (non exposed to pheromone, as such exposure has been shown to lead to an increased size of the corresponding glomerulus (Guerrieri, 2012 #2564)) were carefully dissected in phosphate buffer saline (PBS) and fixed overnight in 4% Electron Microscopy-grade formaldehyde solution in PBS at 4 °C. After rinsing in PBS, the brains were pre-incubated in PBS with 2% Normal Goat Serum (NGS) and 0.5% Triton X 100 and then incubated with the synapsin 1 antibody (1:25 in PBS with 0.5% Triton X and 2% NGS for 5 days at 4 °C). After rinsing, brains were incubated with the secondary antibody (1:250 in PBS with 1% NGS for 3 days at 4 °C; Alexa-Fluor-488-conjugated anti-mouse; Invitrogen, Abingdon, UK). Brains were then rinsed again in PBS, dehydrated in a graded ethanol series, and mounted in methyl salicylate on aluminum slides between two microscopic cover glasses.

### 2.4. Confocal Microscopy

Whole-mount brains were observed and scanned with a Nikon A1 laser confocal microscope. Images were acquired with a Plan Fluor objective (10×/NA 0.3) with an additional 3.5 × digital zoom. Fluorescence was detected with a GFP filter, and images were scanned at 1024 × 1024 pixels, a 4 × frame average, and with a step size of 2 μm. Nikon files were transformed into tiff stacks in Fiji software (Image J, version 2.0.0, National Institutes of Health, Bethesda, ML, USA) and imported into Amira 3.1.1 (Visualization Sciences Group, Mérignac, France).

### 2.5. Reconstructions and Volume Measurements

Outlines of the three glomeruli that constitute the *S. littoralis* MGC were manually traced with the computer mouse in every other section with the “label field” function, as earlier described [14,31], and intermediate section surfaces were interpolated. The “SurfaceGen” tool was used to reconstruct the surface of each glomerulus, and its volume was calculated using the “Measure” tool.

### 2.6. Statistical Analysis

In SSR, odorants were considered as active if the response they elicited was statistically different from the response elicited by the solvent alone (hexane). We tested these differences using one-way ANOVA followed by Tukey’s post hoc test. Volumes of all three MGC glomeruli were normally distributed in both WT and KO males according to a Shapiro-Wilk test (in all cases W > 0.873 and *p* > 0.07); hence, a two-sample *t*-test was used to compare volumes of each MGC glomerulus between KO and WT males using XLSTAT 19.03 (Addinsoft, New York, NY, USA).

## 3. Results

### 3.1. SlitOR5 Knock-Out Specifically Abolishes the Responses of LT1a Neurons to Z9,E11-14:OAc

**Figure 1:**
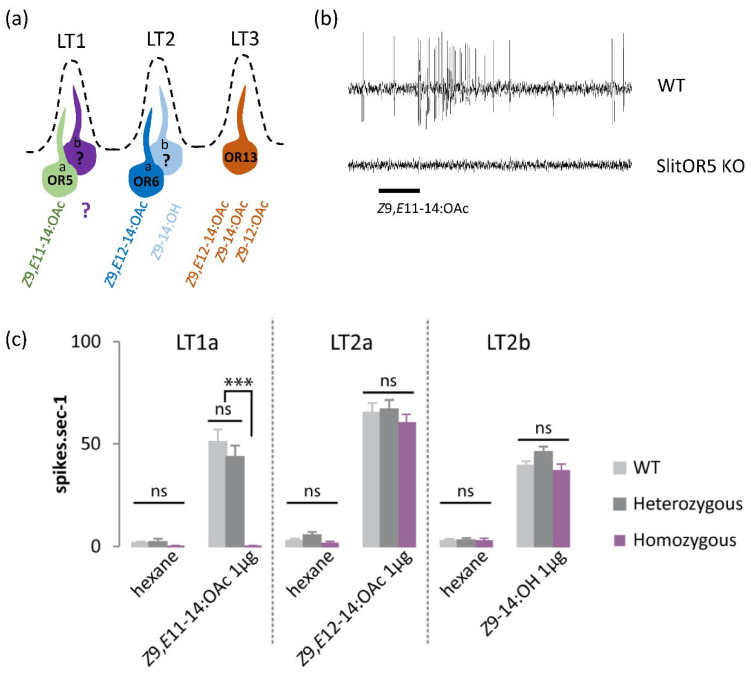
Effects of SlitOR5 knock-out on pheromone reception. (**a**) Diagram showing the different types of long trichoid (LT) sensilla found on *S. littoralis* male antennae, associated olfactory sensory neurons (OSNs a and b), and pheromone compounds detected when known (?: unknown). (**b**) Typical recording traces obtained from LT1a OSNs of wild type (WT) and SlitOR5 homozygous mutant males (KO) of *Spodoptera littoralis* when stimulated with *Z*9,*E*11-14:OAc. LT1a OSNs exhibited no spontaneous activity in SlitOR5 knock-out (KO) individuals, and the response to the major pheromone component was completely abolished. (**c**) Responses to pheromone components (1 µg in the stimulus cartridge) of different pheromone sensitive OSNs (LT1a, LT2a, and LT2b) from wild type males (WT, light gray, *n* = 13), SlitOR5 heterozygous (dark gray, *n* = 12) and homozygous mutant (purple, *n* = 12) males. Responses are measured as action potential frequency (spike·s^−1^) using SSR. Plotted values represent the mean response ± s.e.m (standard error of the mean). *** *p* < 0.001, significantly different from the response of the other genotypes; n.s.: not significantly different (one-way ANOVA, followed by a Tukey’s post hoc test).

*S. littoralis* antennae exhibit three types of pheromone sensitive long trichoid (LT) sensilla named LT1, LT2, and LT3 [28,29,30] (Figure 1a). They are easily recognizable by their morphology, and no morphological difference was obvious between WT and KO adults. LT1 sensilla houses two OSNs, and one of them, LT1a, is tuned to the major pheromone component (*Z*9,*E*11-14:OAc), whereas the ligand for the second OSN is unknown with very low spontaneous activity [28,29]. LT2 also houses two OSNs, with LT2a tuned to the minor pheromone component *Z*9,*E*12-14:OAc, and LT2b tuned to *Z*9-14:OH [28]. LT3 has not been considered in this study since it has only been found at the distal part of the antenna and houses only one OSN tuned to diverse compounds (*Z*9,*E*12-14:OAc, *Z*9-tetradecadienyl acetate: *Z*9-14:OAc, and *Z*9-dodecenyl acetate: *Z*9-12:OAc) [30]. LT1a, LT2a and LT2b OSN types were stimulated with their corresponding activating pheromone components, *Z*9,*E*11-14:OAc, *Z*9,*E*12-14:OAc, and *Z*9-14:OH. Recordings showed equal responses between WT and heterozygote mutant individuals for all the OSNs investigated (Figure 1). These responses were significantly higher (LT1a: df = 8, F = 43.03, *p* = 2 × 10^−16^; LT2a: df = 5, F = 117.7, *p* = 2 × 10^−16^; LT2b: df = 5, F = 110.8, *p* = 2 × 10^−16^) compared to the marginal responses to the solvent, hexane.

Concerning the homozygote mutants, we first noticed that SlitOR5 knock-out completely abolished the spontaneous activity in LT1a OSNs (Figure 1b,c) but not in other OSNs. No significant differences were observed between the WT and the KO individuals for the responses of LT2a and LT2b OSNs to *Z*9,*E*12-14:OAc and *Z*9-14:OH, respectively (df = 2, F = 0.49, *p* = 0.617 and df = 2, F = 2.77, *p* = 0.078) (Figure 1c). On the contrary, LT1a OSNs of homozygote mutants did not respond at all to the major pheromone component, *Z*9,*E*11-14:OAc (Figure 1a,b), whereas LT1a OSNs of WT and heterozygote individuals responded with a very high action potential frequency to this component (Figure 1c). The difference between the homozygotes and WT/heterozygotes was statistically significant (df = 2, F = 31.48, *p* = 1.83 × 10^−8^).

### 3.2. SlitOR5 Knock-Out Modifies the Size of the Cumulus in the Macroglomerular Complex

**Figure 2:**
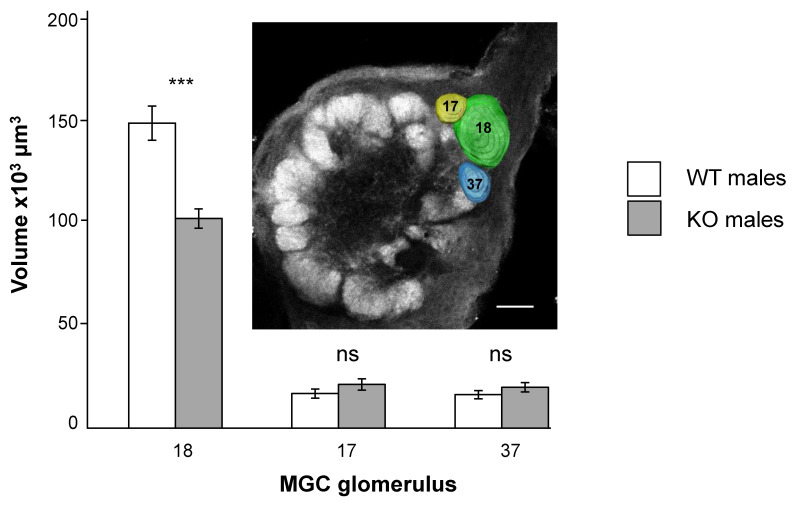
Effects of SlitOR5 knock-out on the macroglomerular (MGC) complex anatomy. Inset: Frontal optical section through the right antennal lobe of an *S. littoralis* mutant (KO) male with superimposed reconstructions of the three macroglomeruli in color: the cumulus (18) and the two minor MGC glomeruli (17 and 37). Glomerulus 18, also named the cumulus, receives input from neurons responding to the major pheromone component, *Z*9,*E*11-14:OAc, detected by SlitOR5. Macrogomerulus volume comparison between wild type (WT, *n* = 12, white bars) and SlitOR5 knock-out (KO, *n* = 12, grey bars) *Spodoptera littoralis* males. Glomerulus 18 is significantly smaller in KO individuals. Bars indicate mean volumes ± s.e.m. Scale bar 50 μm; *** *p* < 0.0001, ns not significant.

*S. littoralis* AL neuroanatomy has been described in detail [32]. Sixty-two glomeruli have been identified and their relative sizes described. Three independent studies on the MGC showed three glomeruli named a, b, and c [33,34] corresponding to 18, 17, and 37, respectively [32]. Glomeruli 17 and 37 have been shown to receive input from the OSNs tuned to the minor pheromone component *Z*9,*E*12-14:OAc, and from the behavioral antagonist *Z*9-14:OH, respectively [34]. Glomerulus 18, also named the cumulus, is the largest one, located at the entrance of the antennal nerve. It has been found to receive input specifically from OSNs tuned to the major pheromone component *Z*9,*E*11-14:OAc, detected by SlitOR5. When comparing volumes of individual MGC glomeruli, the cumulus was significantly smaller in KO males than in WT males (t = 4.86, *p* < 0.0001), whereas the two other MGC glomeruli were not significantly different in size between KO and WT (MGC 17: t = −1.27, *p* = 0.219; MGC 37: t = −1.18, *p* = 0.253) (Figure 2).

## 4. Discussion

Major advances in our understanding of the molecular basis of odorant detection in insects were in part propelled by the global interest in moth sex pheromone communication. Moth PRs were among the very first insect odorant receptors ever characterized, such as those of *Heliothis virescens* [35] and *B. mori* [36,37]. Since then, a large number of moth PRs have been characterized in a variety of species, and most appear to cluster in a specific “PR clade” in the Lepidoptera OR phylogeny [6]. Recently, we identified PRs responsible for the detection of the different components of the *S. littoralis* sex pheromone blend [26,30]. Surprisingly, while the PRs tuned to the minor components (SlitOR6 and SlitOR13) do cluster in the PR clade, SlitOR5—the PR tuned to the main component—belongs to a separate clade of Lepidoptera ORs. This led us to suggest that moth PRs tuned to aliphatic pheromone compounds appeared at least twice during Lepidoptera evolution [26], a suggestion recently confirmed by other studies [38,39]. The functional characterization of such divergent PRs in vivo in relationship with their sequence analyses may help understand how they evolved. In that view, we have generated OR5-knocked-out *S. littoralis* individuals to investigate the function of SlitOR5 in vivo as a complementary strategy to heterologous expression.

We first looked at the SlitOR5 loss-of-function effects at the peripheral level within the antennae. Whereas classical moth PRs could be assigned to trichoid sensilla [5], none of the divergent PRs characterized to date have been assigned to a sensillum type. Using SSR on WT and SlitOR5 KO individuals, we could here unequivocally identify the OSNs that express SlitOR5 in trichoid sensilla. Indeed, recording the activity of the different known pheromone sensitive OSNs one by one and comparing responses of WT and mutant males, we precisely identified LT1a OSNs as responsible for the detection of the main pheromone component (*Z*9,*E*11-14:OAc) via the expression of SlitOR5. Thus, although moth PRs appeared at least twice during evolution, they all seem to be expressed in the same sensillum type.

We then investigated if SlitOR5 loss-of-function would have any impact on AL structure. Indeed, studies on other insects have shown contrasting results on OR/Orco loss-of-function effects on AL anatomy, suggesting diverse scenarii of OR impact on AL development. For instance, the Orco-OR complex plays contrasting roles in glomerulus formation in *Drosophila* versus ants and bees. Mutations in Orco do not affect the development of glomeruli in *Drosophila* [7,23], while Orco mutant ants lack most of the glomeruli found in WT and present a two-third reduced volume of the whole AL [20,21]. In bees, Orco mutants have significantly fewer glomeruli, a smaller volume of the AL, but larger mean individual glomerulus volumes compared to WT [22]. In the flour beetle *Tribolium castaneum*, Orco knock-down via RNAi at the late larval stage resulted in normal AL glomerulization at adult emergence, but in a heavily reduced glomerulization seven days later, whereas the OSNs still locate normally in the antennae at both ages [40]. This led the authors to suggest that Orco is not necessary for the initial formation of AL glomeruli and their maturation during metamorphosis but that OR/Orco driven olfactory activity is necessary for the AL maintenance after adult emergence. In the moth *M. sexta*, Orco disruption resulted in MGC relative volume reduction [19], but the effects on the whole AL were not investigated.

All these studies focused on Orco, and rare are studies conducted on canonical ORs, including PRs. Here, we revealed that the knock-out of a moth PR, SlitOR5, did not impact the number of macroglomeruli in the male MGC but led to the specific volume reduction in the glomerulus 18, involved in *Z*9,*E*11-14:OAc signal integration. This clearly links glomerulus 18 to SlitOR5-expressing OSNs and indicates reduced MGC growth in the absence of the normally expressed PR. In the literature, glomerulus size is usually proposed to be related to OSN activity, OSN axonal branching, and the number of synaptic contacts with AL neurons [14,31,41,42]. For instance, male *S. littoralis* pre-exposure to Z9,E11-14:OAc induces an increased volume of glomerulus 18 [14,31]. The observed SlitOR5 mutant neuroanatomical phenotype may result from reduced neural connectivity between SlitOR5-expressing OSNs and olfactory neurons in the AL (as a result of SlitOR5-OSNs failing to develop properly or dying early in development), as a result of a lack of OSN activity due to the absence of SlitOR5. In *B. mori*, TALEN-mediated loss of the PR tuned to bombykol (BmorOR1) had no effect on the projections of bombykol-sensitive OSNs in the AL [43], suggesting that BmorOR1-KO males develop proper neural connectivity between OSNs and neurons in the AL. However, the branching of individual OSNs has not been analyzed, and the precise size of the macroglomerulus has not been measured in this study. As well, the additional expression of a *Plutella xylostella* moth PR (PxylOR1) in the WT bombykol-sensitive OSNs did not change the projection pattern of these OSNs in the AL [24]. In *Anopheles gambiae*, ectopic expression of an additional OR in many OSNs induces a lack of response to the odor of these OSNs, and this was not due to the death or elimination of neurons [44]. In *Drosophila*, OSNs still project to the same glomerulus after OR gene deletion [45,46]. We notably observed no spontaneous activity in *S. littoralis* LT1a OSN mutants. Similarly, in *H.* *armigera*, knocking out HarmOR16, a receptor for a pheromone antagonist, led to a reduced spontaneous activity level in the OSNs usually expressing HarmOR16 compared to WT [25]. In *B. mori* as well, the level of spontaneous activity of bombykol-sensitive OSNs was largely reduced in BmorOR1 mutants [43]. Again, these observations made in moths match with observations in *Drosophila*: spontaneous activity was greatly reduced in Or67d *Drosophila* mutants [46], Or67d being the receptor to the pheromone cis-vaccenyl acetate.

Taken together, these reports and our data suggest that the observed neuroanatomical *S. littoralis* mutant phenotype represents activity-dependent neuroplasticity, but we can not rule out the possibility of reduced OSN axon projection. Interestingly, the glomerulus 18 was still quite large in KO males and still larger than the two other glomeruli in the MGC. One potential explanation comes from *Drosophila* studies that propose that projection neurons (PNs) also play a role in the glomerular constitution [47,48]. A second potential explanation is that synaptic connections with AL neurons in the cumulus after the loss of SlitOR5 can still occur since Orco is still present in LT1a OSNs. In addition, it is possible that other chemosensory receptors, such as ionotropic receptors (IRs), are expressed in the SlitOR5-OSNs. In *Drosophila* and *Aedes aegypti* it has been demonstrated recently that ORs and IRs can co-express in the same OSN [49,50]. However, we have no evidence yet of such coexpression in other insects. Further experiments, such as the identification of chemoreceptors possibly co-expressed with SlitOR5, visualization of SlitOR5-expressing OSNs within the antennae, and their axon projection into the AL of mutants are still needed to confirm these hypotheses.

## Data Availability

Not applicable.
